# The role of multidisciplinary infection prevention teams in identifying community transmission of SARS-CoV-2 in the United States

**DOI:** 10.1017/ice.2020.360

**Published:** 2020-07-23

**Authors:** Scott J. Crabtree, Stuart H. Cohen

**Affiliations:** 1 Division of Infectious Disease, University of California Davis Medical Center, Sacramento, California; 2 Hospital Epidemiology and Infection Control, University of California Davis Medical Center, Sacramento, California

*To the Editor*—The first case cluster of what would be later called coronavirus disease 2019 (COVID-19) was reported in Wuhan, China, on December 31, 2019.^1^ By January 20, 2020, the first imported case in the United States was identified in a returning traveler.^2^ The first community-transmitted case of COVID-19 was not identified in the United States until February 26, 2020, at the University of California Davis Medical Center (UCDMC) in Sacramento, California, in a patient without known travel to China or contacts with a known patient with COVID-19. Prior to this, the Centers for Disease Control and Prevention (CDC) guidance had recommended SARS-CoV-2 testing only in these patient populations. Through the coordinated efforts of UCD’s multidisciplinary infection prevention (IP) program, the patient was identified as a possible COVID-19 case and obtained SARS-CoV-2 testing.

On February 15, 2020, the case patient presented to a local community hospital with complaints of a flu-like illness. She decompensated shortly after her admission, requiring intubation, vasopressors, and progressively greater ventilatory support. Arrangements were therefore made to have her transferred to UCDMC for the possible initiation of extracorporeal membrane oxygenation for acute respiratory distress syndrome. She arrived at UCDMC on hospital day 5 (HD5). On HD7, UCDMC’s IP team conducted its weekly rounds in the medical intensive care unit.

The IP team is a multidisciplinary team of an infectious diseases (ID)–trained physician, an ID-trained pharmacist, an IP nurse, and a unit nurse champion (Table 1). This team rounds daily in a different ICU with a recurring weekly schedule for individual units. During rounds, each patient is reviewed through the electronic medical record and via discussion with the bedside nurse to evaluate for possible infection prevention and antimicrobial stewardship interventions. Efforts are focused on reducing unnecessary lines and devices, ensuring appropriate use of isolation precautions, and improving antibiotic utilization. Recommendations are given directly to the bedside nurse when applicable or are later directed to the primary physician. At times, patients with complicated, presumed infectious processes are also referred to the Infectious Diseases Consultation Service for further evaluation. Rounds typically require an hour daily, depending on the complexity of the patient population and the size of the unit. These teams have been active at UCDMC since the beginning of 2018 and are considered an important arm of UCDMC’s IP program.

**Table 1.** Infection Prevention (IP) Intensive Care Unit (ICU) Multidisciplinary Team Composition

Member	Role
ID physician	Team lead and rounds facilitator; provides expert guidance on clinical application of IP and ASP concepts
IP nurse	Reviews patients for presence of short-term lines and devices, isolation needs, and *Clostridioides difficile* surveillance; ensures follow up through the unit nurse champion
ID pharmacist	Reviews patients’ antimicrobial regimen for appropriateness and potential for optimization
Unit nurse champion	Serves as a liaison for the IP team and the nursing personnel in the unit; ensures follow up of IP ICU team’s recommendations

Note. ID, infectious diseases; ASP, antimicrobial stewardship program.

At this point, the patient remained intermittently febrile but stable on the ventilator with an improving PaO_2_/FiO_2_ and minimal respiratory secretions. Laboratory testing was remarkable, with a white blood cell count of 8.0 cells/mm^3^ (2.5% lymphocytes), sodium of 126 mmol/L, and worsening creatinine of 1.89 mg/dL. Computed tomography images of the chest showed confluent consolidative and ground glass opacities in the right upper and (to lesser extent) middle lobes. Testing for common respiratory pathogens was negative. She had been in good health prior to her illness, with no significant travel or exposure histories. The patient’s case was discussed with her bedside nurse, who confirmed that SARS-CoV-2 was considered by her primary team, but given the absence of exposures, testing for this agent was not pursued. We then made the decision for the bedside nurse to further clarify patient’s occupational, travel, and potential exposure histories with her family, with plans for the IP team to reassess later that morning.

The patient’s bedside nurse subsequently reported that the patient worked in the service industry and had had direct and close interaction with multiple individuals on a daily basis. One of these individuals had returned from China a few weeks prior and was briefly detained by customs upon arrival. No further details of this encounter were available. The community in which she worked was located southwest of Sacramento near a local Air Force base, where a number of diplomatic evacuees had been in recent quarantine. We then elected to review her case with the Director of Hospital Epidemiology and Infection Control, and we collectively decided that, despite the absence of clear exposure risks, given her clinical picture and its unknown cause, testing for SARS-CoV-2 would be requested through the county public health officer. This request was first denied due to the patient’s not meeting the CDC’s criteria for a person under investigation (PUI), but 2 days later (on HD9) was granted by the CDC. On HD13, nasopharyngeal RT-PCR results returned positive for SARS-CoV-2. Due to ongoing critical illness, the ID consultation service made a request to the Food and Drug Administration for compassionate-use remdesivir, which was granted that same day. The first dose was administered on HD14. On HD19, the patient was extubated, and on HD31 she was discharged home.

Antimicrobial stewardship “handshake” rounds, involving the regular in-person interaction between stewardship teams and frontline providers, were first rolled out at Children’s Hospital Colorado in 2013, with good results.^3^ Such rounds have been associated with sustained improvements in antibiotic utilization,^4^ high critical-care physician satisfaction,^5^ and improved and timely ID consultation.^6^ Similar IP-focused multidisciplinary teams have been shown to reduce the rate of catheter-associated urinary tract infections and central-line–associated bloodstream infections.^7^ However, given that this strategy is only a recent development, further research is needed to better appreciate its impact and to optimize this practice. This case highlights an additional and critical surveillance role that a multidisciplinary IP team can provide, especially in times of emerging infectious disease. Due to the identification of this case, the CDC reviewed and later revised its PUI case definition, with widespread impact on the management of the COVID-19 epidemic within the United States.^8^
